# ROS-Induced SIRT2 Upregulation Contributes to Cisplatin Sensitivity in Ovarian Cancer

**DOI:** 10.3390/antiox9111137

**Published:** 2020-11-16

**Authors:** Wenyu Wang, Jihye Im, Soochi Kim, Suin Jang, Youngjin Han, Kyung-Min Yang, Seong-Jin Kim, Danny N. Dhanasekaran, Yong Sang Song

**Affiliations:** 1 Interdisciplinary Program in Cancer Biology, Seoul National University College of Medicine, Seoul 03080, Korea; wywang@snu.ac.kr; 2 Cancer Research Institute, Seoul National University College of Medicine, Seoul 03080, Korea; imjirody@gmail.com (J.I.); suinjang213@gmail.com (S.J.); youngjin.han@snu.ac.kr (Y.H.); 3 WCU Biomodulation, Department of Agricultural Biotechnology, Seoul National University, Seoul 03080, Korea; 4 Department of Neurology and Neurological Sciences, Stanford University, School of Medicine, Stanford, CA 94305-5101, USA; skim245@stanford.edu; 5 Precision Medicine Research Center, Advanced Institute of Convergence Technology, Seoul National University, Suwon, Gyeonggi-do 16229, Korea; yangkm@snu.ac.kr (K.-M.Y.); jasonsjkim@snu.ac.kr (S.-J.K.); 6 Department of Transdisciplinary Studies, Graduate School of Convergence Science and Technology, Seoul National University, Suwon, Gyeonggi-do 16229, Korea; 7 MedPacto Inc., 92, Myeongdal-ro, Seocho-gu, Seoul 06668, Korea; 8 Stephenson Cancer Center, The University of Oklahoma Health Sciences Center, Oklahoma City, OK 73104, USA; Danny-Dhanasekaran@ouhsc.edu; 9 Department of Cell Biology, The University of Oklahoma Health Sciences Center, Oklahoma City, OK 73104, USA; 10 Department of Obstetrics and Gynecology, Seoul National University College of Medicine, Seoul 03080, Korea

**Keywords:** ovarian cancer, SIRT2, oxidative stress, ROS, cisplatin, chemoresistance

## Abstract

Cisplatin resistance remains a significant obstacle for improving the clinical outcome of ovarian cancer patients. Recent studies have demonstrated that cisplatin is an important inducer of intracellullar reactive oxygen species (ROS), triggering cancer cell death. Sirtuin 2 (SIRT2), a member of class III NAD^+^ dependent histone deacetylases (HDACs), has been reported to be involved in regulating cancer hallmarks including drug response. In this study, we aimed to identify the role of SIRT2 in oxidative stress and cisplatin response in cancer. Two ovarian cancer cell lines featuring different sensitivities to cisplatin were used in this study. We found different expression patterns of SIRT2 in cisplatin-sensitive (A2780/S) and cisplatin-resistant (A2780/CP) cancer cells with cisplatin treatment, where SIRT2 expression was augmented only in A2780/S cells. Furthermore, cisplatin-induced ROS generation was responsible for the upregulation of SIRT2 in A2780/S cells, whereas overexpression of SIRT2 significantly enhanced the sensitivity of cisplatin-resistant counterpart cells to cisplatin. Our study proposes that targeting SIRT2 may provide new strategies to potentiate platinum-based chemotherapy in ovarian cancer patients.

## 1. Introduction

Ovarian cancer is the most lethal gynecologic cancer in women worldwide [1]. Most patients are diagnosed at advanced stages largely due to the absence of specific symptoms and lack of early diagnostic markers [2,3]. The standard treatment of ovarian cancer currently is debulking surgery followed by platinum-based chemotherapy. However, up to 70% of patients experience relapses as a result of chemoresistance [4]. There has only been a very modest improvement in the survival of ovarian cancer patients over the past decades, with the five-year survival rate under 45% [5]. Thus, understanding cisplatin resistance mechanisms is of great significance for improving the clinical outcome of ovarian cancer patients.

Reactive oxygen species (ROS) constitute a group of highly reactive molecules involved in various biological processes [6]. ROS can influence various cancer hallmarks such as cell proliferation, apoptosis, chemoresistance, and act as a double-edged sword in cancer [7,8]. Cisplatin is widely used in the treatment of ovarian cancer and has been demonstrated to induce ROS generation [9]. It was reported that cisplatin could induce intrinsic and ROS-dependent apoptosis in ovarian cancer [10]. Our previous study demonstrated that cisplatin resistance was caused by mitochondrial fission under hypoxic conditions via ROS in ovarian cancer cells [11]. Acquired chemoresistance that arises after long-term exposure to anticancer therapeutics might be multifactorial. The redox homeostasis has been shown to be closely related to chemoresistance [11].

Sirtuins (SIRTs) are class III histone deacetylases (HDACs), which include a family of seven proteins (SIRT1-7) with homology to the silent information regulator 2 (Sir2) gene in *Saccharomyces cerevisiae* in mammals [12,13,14]. SIRTs can deacetylate both histones and nonhistone proteins dependent on nicotinamide adenine dinucleotide (NAD) as a cofactor [15,16]. A great body of evidence has shown that SIRTs are involved in divergent biological processes and play an important role in carcinogenesis and cancer progression [17,18,19,20]. The SIRT family proteins are different in subcellular localization with SIRT1, SIRT6, and SIRT7 in the nucleus, Sirtuin 2 (SIRT2) in the cytosol, and SIRT3, SIRT4, and SIRT5 principally in the mitochondria. Heterogeneous subcellular locations also reflect their various biological functions [21,22]. SIRT2 is predominately localized in the cytoplasm but can translocate to the nucleus during the G2/M cell cycle transition. SIRT2 is widely expressed in different organs and tissues, exerting critical functions in cancer [23]. However, it is still under debate whether SIRT2 is an oncogene or a tumor suppressor. For example, SIRT2 was reported to be downregulated in liver cancer tissues as compared with normal tissues, suggesting its possible role as a tumor suppressor [24]. At the same time, some studies have shown that SIRT2 expression was relatively higher in cancer tissues and that this was positively related to increased microscopic vascular invasion and poor prognosis as an oncogene [25,26]. Researches have shown that SIRT2 deacetylation was actively involved in antioxidant- and redox-mediated cellular homeostasis [14]. Recently, the regulatory function of SIRT2 in drug response has gained attraction. It was demonstrated that SIRT2 could antagonize the cytotoxicity of lapatinib in nasopharyngeal carcinoma [27]. However, the role of SIRT2 in response to cisplatin in ovarian cancer cells remains largely unknown.

In this study, we investigated the differential regulation of SIRT2 expression in response to cisplatin treatment in A2780/S and A2780/CP ovarian cancer cell lines. We found that cisplatin-induced ROS generation was responsible for the upregulation of SIRT2 in A2780/CP cells. Furthermore, overexpression of SIRT2 significantly increased the level of cisplatin-induced apoptosis in A2780/CP cells. Our results have provided new insights into potential therapeutic strategies to overcome cisplatin resistance in ovarian cancer.

## 2. Materials and Methods

### 2.1. Cell Culture

Human ovarian cancer cell line A2780/S and its cisplatin-resistant subline A2780/CP were provided by Professor Benjamin K. Tsang (University of Ottawa, ON, Canada) [28]. The A2780/S and A2780/CP cells were cultured in RPMI 1640 (WelGENE, Seoul, South Korea) supplemented with 10% fetal bovine serum (FBS; WelGENE, Seoul, South Korea). Cisplatin (1 μM) was added to the culture media every other passage to maintain the cisplatin resistance of A2780/CP cells.

### 2.2. MTT Assay

Cell viability was determined using MTT Assay (Amresco, Solon, OH, USA), according to the manufacturer’s instructions. The A2780/S and A2780/CP cells were seeded in 96-well plates, and then cultured with different treatments. The MTT solution was added to each well without discarding culture media. Then, cells were incubated at 37 °C for 3 h. DMSO was added after discarding culture media to dissolve formazan crystals. After incubation on an orbital shaker at room temperature for 30 min, the optical density of each sample was detected at 540 nm using a Multi-Scan Spectrum (Thermo Scientific, Hudson, NH, USA).

### 2.3. Cell Apoptosis Assay

The A2780/S and A2780/CP cells were collected and subjected to Annexin V staining using an FITC-conjugated Annexin V Apoptosis Detection Kit I (BD Pharmingen, CA, USA). Then, proportions of apoptotic cells in each treatment condition were analyzed using a BD FACS Canto II flow cytometer (FACS Canto, BD Biosciences, North Ryde, Australia), according to the manufacturer’s instructions.

### 2.4. Rreactive Oxygen Species (ROS) Level Detection

ROS levels were detected using 2,7-dichloro-dihydrofluorescein diacetate (DCFH-DA) (Sigma-Aldrich, St. Louis, MO, USA) and dihydroethidium (DHE) (Sigma-Aldrich). Cells were harvested and incubated with DHE (5 μM) for 10 min or DCFH-DA (10 μM) for 30 min at 37 °C in the dark. Relative DCF or DHE fluorescence was measured using a BD FACS Canto II flow cytometer (BD Bioscience, North Ryde, Australia).

### 2.5. RNA Extraction and Quantitative Real-Time PCR

Total RNA was extracted from the A2780/S cells and A2780/CP cells using RNAiso Plus reagent (Takara, Tokyo, Japan).

Then, 1 μg of RNA was reverse transcribed into cDNA with the PrimeScript Reverse Transcriptase (Takara, Tokyo, Japan). Quantitative real-time PCR was performed using a QuantiSpeed SYBR Kit (Phile Korea, South Korea), according to the manufacturer’s instructions. Relative expression was evaluated by the 2^−ΔΔCt^ method with GAPDH as an endogenous control. Primer sequences were listed as follows: SIRT2 forward 5′-TCC ACC AAG TCC TCC TGT TC-3′, SIRT2 reverse 5′-TGA AGG ACA AGG GGC TAC TC-3′ and GAPDH forward 5′-GAG TCA ACG GAT TTG GTC GT-3′, reverse 5′-TTG ATT TTG GAG GGA TCT CG-3′.

### 2.6. Western Blotting

Western blotting assay was performed, as previously described [11]. Briefly, cells were harvested and lysed with lysis buffer. The supernatant was collected after centrifugation. Protein concentration was determined using a BCA Protein Assay Kit (Thermo Scientific, Waltham, MA, USA). Proteins were separated by SDS-PAGE and transferred to nitrocellulose membranes. Membranes were blocked with skim milk (5%) in TBS-T (TBS Tween-20 0.1%), and then incubated with specific primary and secondary antibodies. Signals were visualized using a Westsave Western Blotting Detection Kit (AbFrontier, Seoul, South Korea).

### 2.7. Cell Transfection

FLAG-tagged human SIRT2 overexpression plasmids were obtained from Professor Yong-Ho Ahn (Yonsei University, South Korea) [29]. The A2780/CP cells were transfected with FLAG-tagged SIRT2 plasmids or negative control plasmids using Lipofectamine 3000™ reagent (Invitrogen, Carlsbad, CA, USA). Culture media was changed 6 h post transfection.

### 2.8. Statistical Analysis

Data were presented as means ± SEM based on triplicate experiments. Student’s *t*-test and one-way ANOVA analysis with Bonferroni’s post hoc test was performed for comparisons between two groups or groups more than two. Statistical analysis was carried out using GraphPad Prism 7 with the *p* value < 0.05 considered as statistically significant (* *p* < 0.05, ** *p* < 0.01, and *** *p* < 0.001).

## 3. Results

### 3.1. A2780/S Cells and A2780/CP Cells Exhibited Different Sensitivity to Cisplatin

**Figure 1:**
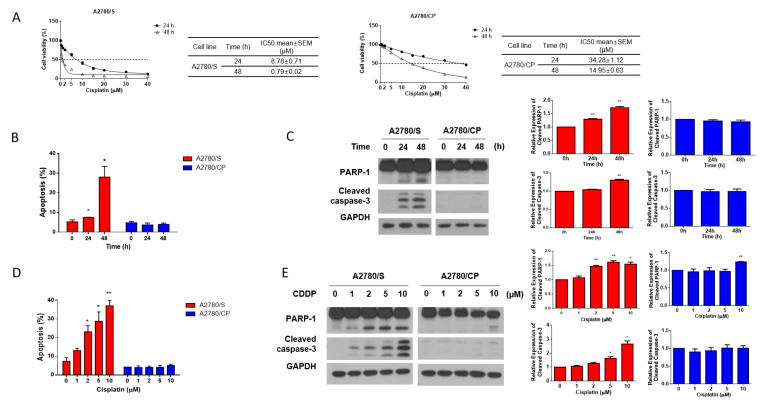
(**A**) A2780/S and A2780/CP cells were treated with the indicated concentrations of cisplatin for 24 and 48 h. Cell viability was evaluated by MTT assay and the best-fit curves were generated to determine IC50 values of A2780/S and A2780/CP cells; (**B**) A2780/S and A2780/CP cells were treated with cisplatin (2 μM) for 24 and 48 h. Cell apoptosis was detected by flow cytometry using Annexin V staining; (**C**) The expression of apoptosis-related proteins was determined by Western blotting; (**D**) A2780/S and A2780/CP cells were treated with cisplatin in different concentrations (0 to 10 μM) for 48 h. Cell apoptosis was detected by flow cytometry using Annexin V staining; (**E**) The expression of apoptosis-related proteins was determined by Western blotting. Results are shown as mean ± SEM (* *p* ≤ 0.05, ** *p* ≤ 0.01).

To explore the underlying mechanisms of cisplatin resistance in ovarian cancer, in this study, we used two human ovarian cancer cell line, i.e., A2780/S cells and its cisplatin-resistant derivative A2780/CP cells. The results of the cell viability assay showed that A2780/CP cells exhibited significantly higher cisplatin resistance than A2780/S cells (Figure 1A). Consistently, cisplatin induced the apoptosis of A2780/S cells in a time- and dose-dependent manner, whereas it showed a very modest impact on the apoptosis of A2780/CP cells (Figure 1B–E). These results indicated that A2780/S cells and A2780/CP cells exhibited different cisplatin sensitivity and could work as a fine model for further study.

### 3.2. Cisplatin Induced ROS Generation in A2780/S Cells

**Figure 2:**
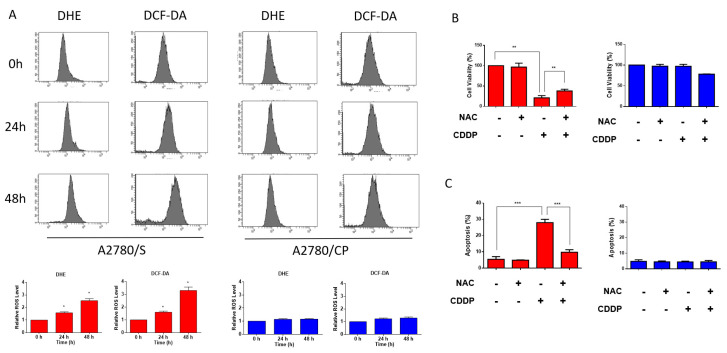
The A2780/S and A2780/CP cells were treated with cisplatin (2 μM) for 24 and 48 h. The intracellular reactive oxygen species (ROS) levels were measured through staining by flow cytometric assay using dihydroethidium (DHE) (**A**) and 2,7-dichloro-dihydrofluorescein diacetate (DCFH-DA) (**B**) staining. The A2780/S and A2780/CP cells were treated with cisplatin (2 μM) only or cotreated with NAC. Cell viability was accessed using an MTT assay (**B**) and cell apoptosis was determined using flow cytometry (**C**). Results are shown as mean ± SEM (* *p* ≤ 0.05, ** *p* ≤ 0.01, *** *p* ≤ 0.001).

Previous researches have demonstrated that the cytotoxic effect of cisplatin was related to the increasing intracellular ROS level [9]. Therefore, we measured the intracellular ROS generation in A2780/S and A2780/CP cells using flow cytometry. It showed that intracellular ROS levels were significantly augmented in A2780/S cells, while those in A2780/CP cells were not affected with the treatment of cisplatin for 24 and 48 h (Figure 2A). Moreover, cisplatin-induced cell death was reduced only in A2780/S cells when cells were co-treated with NAC, a ROS scavenger (Figure 2B,C).

### 3.3. Sirtuin 2 (SIRT2) Might Be a Potential Tumor Suppressor in Ovarian Cancer

**Figure 3:**
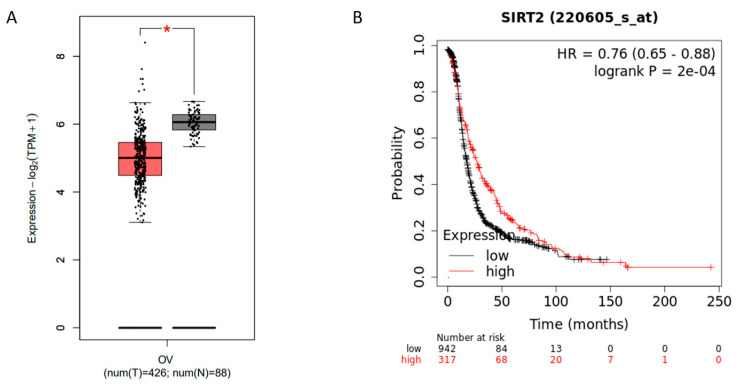
Comparison of Sirtuin 2 (SIRT2) expression in normal ovarian tissues (gray) and ovarian cancer tissues (red) by GEPIA (http://gepia.cancer-pku.cn/index.html) (**A**) (* *p* ≤ 0.05). Progression-free survival analysis based on SIRT2 expression in ovarian cancer patients by Kaplan–Meier Plotter (https://kmplot.com/analysis/) (**B**).

SIRT2 belongs to the sirtuin family and is mainly localized in the cytoplasm. It has been demonstrated to play fundamental roles in cell differentiation, oxidative response, cancer development, etc. However, the role of SIRT2 seems to be controversial as it has been shown to exert both oncogenic or tumor-suppressive functions during different biological processes [23,24,25,26]. To explore the potential role of SIRT2 in ovarian cancer cells, we first analyzed the SIRT2 expression using public databases. Notably, SIRT2 was markedly downregulated in ovarian cancer tissues as compared with normal tissues (Figure 3A). Additionally, higher SIRT2 expression was correlated with longer progression-free survival (PFS) in ovarian cancer patients with platinum-included chemotherapy (Figure 3B). These data suggested that SIRT2 might act as a tumor suppressor and be related to cisplatin sensitivity in ovarian cancer.

### 3.4. SIRT2 Was Upregulated with Cisplatin Treatment in A2780/S Cells

**Figure 4:**
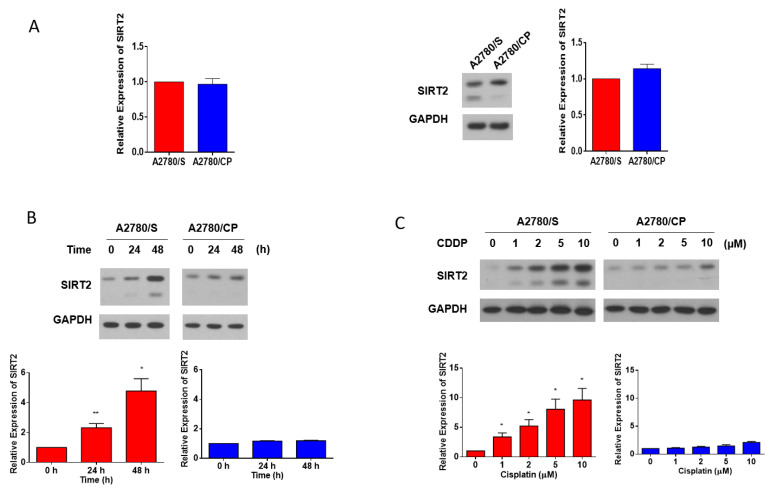
SIRT2 basal expression in the A2780/S and A2780/CP cells were evaluated at the mRNA level and protein level (**A**). SIRT2 expression was detected in the A2780/S and A2780/CP cells with cisplatin treatment (2 μM) for 24 and 48 h (**B**). The expression of SIRT2 was detected in the A2780/S and A2780/CP cells with cisplatin treatment in different concentrations (**C**). Results are shown as mean ± SEM (* *p* ≤ 0.05, ** *p* ≤ 0.01).

In order to identify the potential relationship between SIRT2 expression and cisplatin sensitivity, we detected the basal level of SIRT2 in A2780/S and A2780/CP cells. However, SIRT2 showed no significant differences both at the mRNA and protein levels in these two cell lines (Figure 4A). Considering that SIRT2 is a stress-relevant protein, then, we investigated its expression with the treatment of cisplatin. Intriguingly, SIRT2 expression was dramatically enhanced in cisplatin-treated A2780/S cells in a time- and dose-dependent manner while it showed a very modest change in A2780/CP cells (Figure 4B,C). These data implied that the upregulation of SIRT2 with cisplatin treatment could be associated with cisplatin responsiveness in ovarian cancer cells.

### 3.5. Cisplatin-Induced ROS Generation Was Responsible for SIRT2 Upregulation in A2780/S Cells

**Figure 5:**
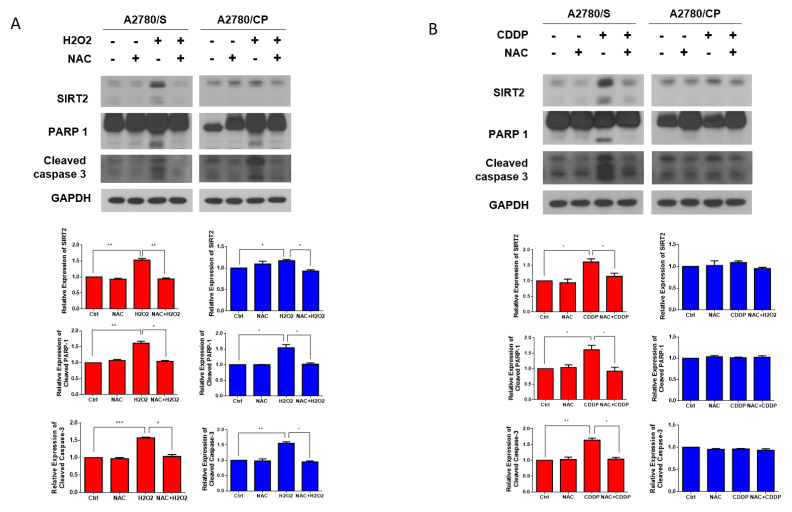
The A2780/S and A2780/CP cells were pretreated with NAC (5 mM) for 1 h, followed by H_2_O_2_ (10 μM) for 48 h. SIRT2 and apoptosis-related proteins were detected by Western blotting (**A**). The A2780/S and A2780/CP cells were pretreated with NAC (5 mM) for 1 h, followed by cisplatin (2 μM) for 48 h. SIRT2 and apoptosis-related proteins were detected by Western blotting (**B**). Results are shown as mean ± SEM (* *p* ≤ 0.05, ** *p* ≤ 0.01, *** *p* ≤ 0.001).

On the basis of the results above, we, then, hypothesized that SIRT2 upregulation in cisplatin-treated cells might be caused by the cisplatin-induced ROS. To validate our hypothesis, first, we treated the A2780/S and A2780/CP cells with hydrogen peroxide (H_2_O_2_) to mimic ROS generation or NAC to scavenge ROS. SIRT2 expression was upregulated in both cell lines in the H2O2 treatment group with a more obvious trend in A2780/S cells, which was reversed by the cotreatment of NAC. In addition, as expected, H2O2 treatment induced the apoptosis of both cells and NAC cotreatment abrogated this effect (Figure 5A). However, cisplatin treatment only resulted in the SIRT2 upregulation and apoptosis in A2780/S cells but not in A2780/CP cells (Figure 5B). This is consistent with the previous results that cisplatin treatment could only significantly induce ROS generation in A2780/S cells.

### 3.6. Overexpression of SIRT2 Enhanced Cisplatin-Induced Apoptosis in A2780/CP Cells

**Figure 6:**
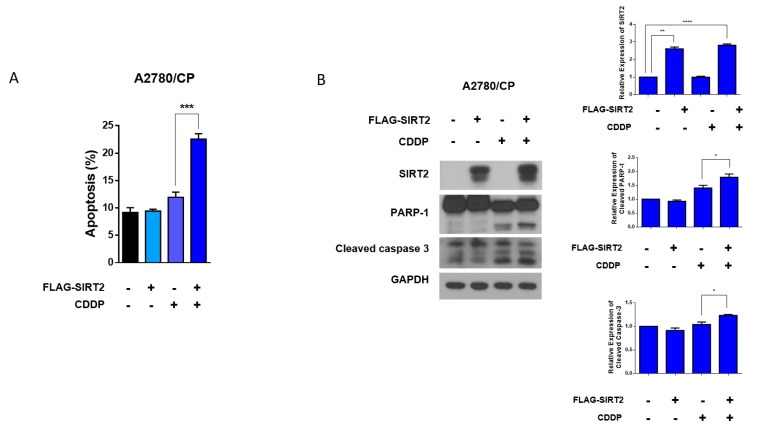
A2780/CP cells were transfected with FLAG-SIRT2 overexpression plasmid or negative control plasmid (5 μg) for 24 h, followed by cisplatin treatment (10 μM) for 48 h. Cell apoptosis was evaluated by flow cytometry (**A**) and Western blotting (**B**). Results are shown as mean ± SEM (* *p* ≤ 0.05, ** *p* ≤ 0.01, *** *p* ≤ 0.001, **** *p* ≤ 0. 0001).

To further explore the role of SIRT2 in cisplatin sensitivity of ovarian cancer cells, next, we transfected FLAG-SIRT2 into A2780/CP cells to transiently overexpress SIRT2. Interestingly, SIRT2 overexpression did not affect the basal apoptosis level of A2780/CP cells but reversed the resistance of A2780/CP cells to cisplatin. Overexpression of SIRT2 significantly promoted the apoptotic cell death of A2780/CP cells treated with cisplatin, as indicated by the increased proportions of apoptotic cells and the cleavages of PARP-1 and caspase-3 (Figure 6A,B). These results implicated that SIRT2 induction could determine cisplatin sensitivity in ovarian cancer cells.

## 4. Discussion

Currently, the standard of care for ovarian cancer is the optimal cytoreductive surgery combined with platinum-based chemotherapy. Despite the high response rates initially, most patients develop acquired chemoresistance leading to relapses [4,5]. Moreover, sensitivity to platinum-based chemotherapies declines with each subsequent relapse [30]. As such, overcoming chemoresistance is one of the most significant obstacles for improving the survival of ovarian cancer patients. Cisplatin is widely used in the treatment of various cancer types [31]. Cisplatin is able to cross-link with DNA forming DNA adducts, which results in DNA damages triggering cell cycle arrest and cell apoptosis [32]. Recent studies have also revealed that cisplatin could induce ROS generation that markedly enhanced the cytotoxicity by nuclear DNA damage. The contribution of cisplatin-induced ROS in cytotoxicity varied among cell types, cell redox status, and bioenergetic functions, etc. [9]. As shown in our results, cisplatin significantly induced the ROS generation in cisplatin-sensitive cells, whereas less significant effects were observed in cisplatin-resistant cells. This suggested that oxidative stress response may play a critical role in the sensitivity to cisplatin of ovarian cancer cells.

SIRTs belong to the class III histone deacetylase family, including seven proteins (SIRT1-7) in mammalian animals. SIRTs share homology with the yeast deacetylase Sir2 but are different in the N- and C-terminal domains [12]. The subcellular localization of SIRTs is restricted in the nucleus, cytoplasm, and mitochondria. SIRT2 is the only member predominantly located in the cell cytoplasm but is also found to regulate the cell cycle in the nucleus [33]. The activity and expression of SIRT2 are altered depending on the metabolism of cells where activated in low-energy status while inhibited in high-energy status [14]. SIRT2 has been shown to play a critical role in cancer but it is still inconsistent whether it is an oncogene or a tumor suppressor. In breast cancer, lower SIRT2 expression was detected in cancer tissues as compared with adjacent normal tissues [24,34]. In addition, SIRT2 was also downregulated in metastatic sites as compared with primary sites [24]. Similarly, McGlynn et al. investigated that SIRT2 transcripts were significantly lower in malignant breast tissues than non-malignant or normal counterparts. However, they further observed that SIRT2 nuclear expression was negatively associated with patient prognosis [35]. Taken together, these results suggest that SIRT2 might serve as a tumor suppressor in carcinogenesis but as an oncogene during tumor development. In lung cancer, it has been demonstrated that SIRT2 was significantly downregulated in tumor tissues as compared with paired adjacent normal tissues at the mRNA and protein levels. Further experiments have shown that SIRT2 could inhibit cell proliferation, increase apoptosis, and induce cell cycle arrest both in vitro and in vivo [36,37]. On the contrary, Grbesa et al. showed that SIRT2 expression was markedly higher in tumor tissues as compared with in normal tissues, and higher SIRT2 levels were related to shorter recurrence-free survival [38]. These conflicting results indicate that the regulatory mechanisms of SIRT2 in cancer are multiple and complicated and warrant further elucidation.

Du et al. were the first to investigate the expression and function of SIRT2 in ovarian cancer. They found that SIRT2 was significantly downregulated in ovarian cancer tissues and cell lines as compared with normal epithelium counterparts. Furthermore, SIRT2 could reduce the proliferation, migration, and invasion, serving as a tumor suppressor [39]. In the present study, we also analyzed SIRT2 expression through public databases. SIRT2 showed a lower expression level in tumor tissues than normal tissues in ovarian cancer. We further analyzed the relation between SIRT2 expression and patient clinical outcomes. Intriguingly, we found that low SIRT2 expression was associated with shorter PFS in ovarian cancer patients receiving platinum-included chemotherapy. This prompted us to look into the expression difference between cisplatin-sensitive and -resistant cell lines. However, A2780/S and A2780/CP cells showed no significant difference in SIRT2 expression at the basal level. Given that the expression and activity of SIRT2 may vary significantly when cells are stimulated with stress, then, we investigated the expression of both cell lines when treated with cisplatin. It is worth noting that SIRT2 was markedly upregulated with the treatment of cisplatin in a time- and dose-dependent manner only in A2780/S cells, while a very modest alteration of SIRT2 was seen in A2780/CP cells. This suggested that SIRT2 could be a critical biomarker for indicating the response to cisplatin in ovarian cancer. Combining previous results that cisplatin only significantly induced ROS generation in A2780/S cells, we hypothesized that cisplatin-induced ROS might be responsible for the upregulation of SIRT2 in A2780/S cells. Then, we treated H_2_O_2_ to mimic ROS generation and NAC to scavenge ROS in both cell lines. We found that H_2_O_2_ treatment could induce SIRT2 upregulation and apoptosis in both cell lines with a more distinct tendency in A2780/S cells, while this effect could be reversed by NAC cotreatment. Consistent with our result, Wang et al. also reported that SIRT2 was upregulated by oxidative stress induced by H_2_O_2_ treatment, which could promote cell death caused by H_2_O_2_ [40]. These results suggested that ROS generation was an important inducer that triggered SIRT2 upregulation, which also explained why cisplatin induced SIRT2 upregulation only in A2780/S cells. Next, in order to study the function of SIRT2 in cisplatin response, we overexpressed SIRT2 in A2780/CP cells. Overexpression of SIRT2 significantly enhanced the cisplatin-induced cell death in A2780/CP cells. In other words, SIRT2 seemed to have resensitized cisplatin-resistant cells. To our knowledge, for the first time, we have demonstrated that SIRT2 could contribute to cisplatin sensitivity. Previously, SIRT2 was reported to increase the resistance of anticancer drugs in melanoma and nasopharyngeal carcinoma [27,41]. These results seem contradictory but also make great sense in view of the dual tumor suppressive and oncogenic role of SIRT2 in cancer.

Cancer is a highly heterogeneous disease and the biological features and behaviors of cancers are largely dependent on the tumor microenvironment [42]. The different regulatory roles of SIRT2 in drug response might be due in part to the different substrates and downstream pathways. Consistent with previously mentioned publications, our results showed that SIRT2 was predominantly localized in the cytoplasm but also existed in the nucleus in ovarian cancer cells (Figure S1C). In accordance with its subcellular location, SIRT2 is able to deacetylate plenty of nonhistone proteins, such as CDK9, PGAM2, Par-3, G6PD, FOXO1, FOXO3, etc. in the cytoplasm while it can deacetylate lysine 18 on histone H3 (H3-K18Ac), lysine 56 on histone H3 (H3-K56Ac), and lysine 16 on histone H4 (H4-K16Ac) in the nucleus [24,43,44,45,46,47,48,49,50]. Therefore, we would like to go deeper into the underlying mechanism of how SIRT2 regulates chemosensitivity in ovarian cancer later on. Our pilot experiment results showed that FOXO3, one of the substrates of SIRT2, was significantly downregulated only in A2780/S cells when treated with cisplatin in a time- and dose-dependent manner, while little change could be observed in A2780/CP cells (Figure S1A,B). As an important transcription factor, FOXO3 was mainly located in the nucleus but could also be detected in the cytoplasm (Figure S1C). A negative correlation could be roughly observed between the expression of FOXO3 and SIRT2. These results provided a prospective clue that SIRT2 might influence cisplatin response through deacetylating FOXO3 and altering downstream pathways, which are considered to be included in our further study. FOXO3 also plays a dual oncogenic and tumor-suppressive role in cancer, therefore, multiple competing mechanisms should be taken into consideration in future study [51].

## 5. Conclusions

Our study, for the first time, identified the critical role of SIRT2 in the cisplatin response in ovarian cancer. Cisplatin could induce ROS generation resulting in the upregulation of SIRT2 in cisplatin-sensitive cells. Overexpression of SIRT2 in cisplatin-resistant cells could resensitize these cells to cisplatin, enhancing cisplatin-induced cell death. Our study sheds new light on novel strategies for overcoming cisplatin resistance in ovarian cancer, whereas further research elucidating the mechanism underneath is warranted.

## Supplementary Materials

The following are available online at https://www.mdpi.com/2076-3921/9/11/1137/s1, Figure S1: FOXO3 might be a downstream target of SIRT2 and contributes to cisplatin sensitivity in ovarian cancer.
