# Supplementary material for: ROS-Induced SIRT2 Upregulation Contributes to Cisplatin Sensitivity in Ovarian Cancer
Source: Antioxidants (Basel). 2020 Nov 16;9(11):1137. doi: 10.3390/antiox9111137 (PMC7698236; doi:10.3390/antiox9111137)
Supplement: Supplementary file 1 [file antioxidants-09-01137-s001.pdf]

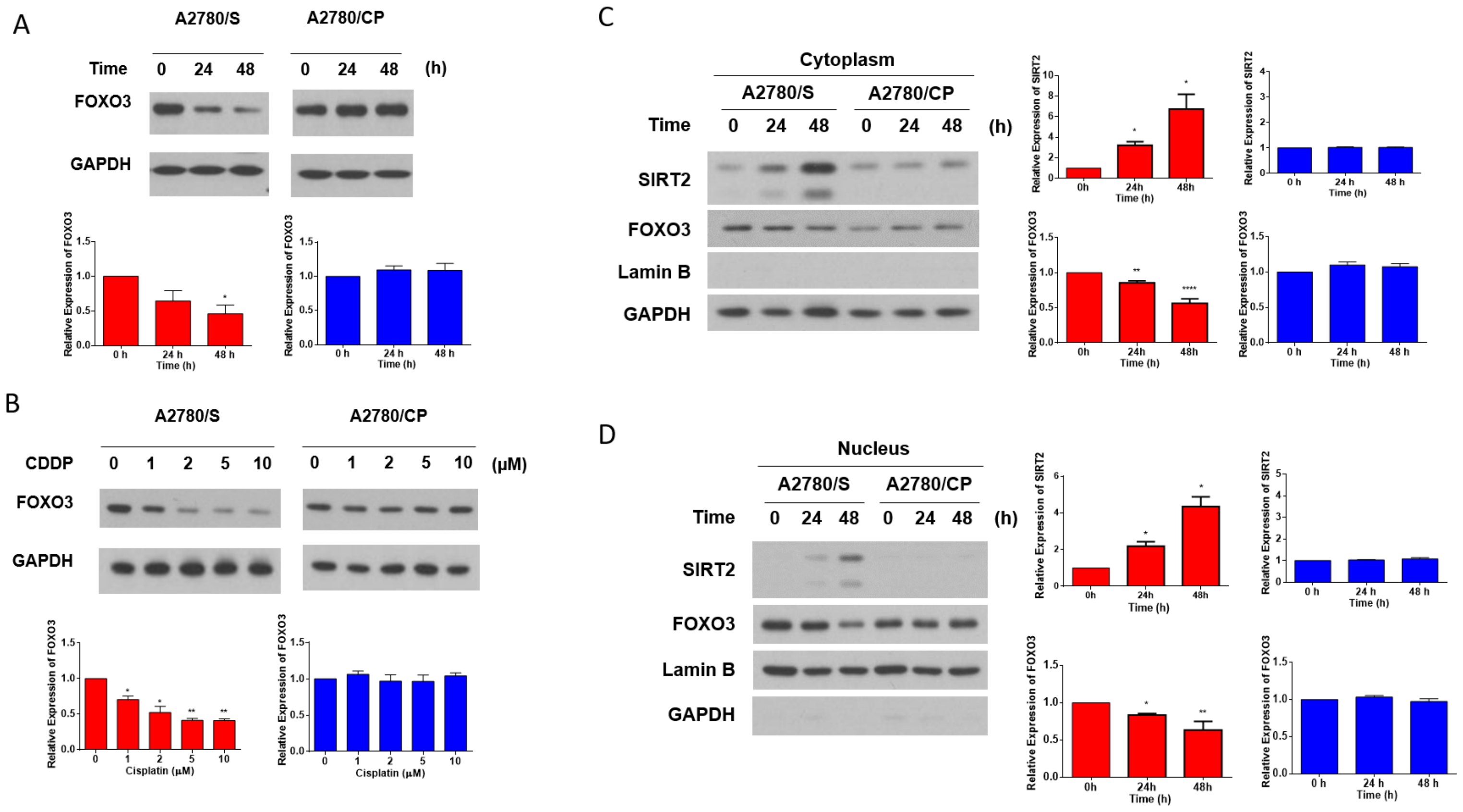

Figure S1. FOXO3 might be a downstream target of SIRT2 and contributes to cisplatin sensitivity in ovarian cancer. FOXO3 expression was detected in A2780/S and A2780/CP cells with cisplatin treatment (2 μM) for 24 and 48 h (Figure S1A). FOXO3 expression was detected in A2780/S and A2780/CP cells with cisplatin treatment in different concentrations (Figure S1B). FOXO3 expression in cytoplasm and nucleus level was detected in A2780/S and A2780/CP cells with cisplatin treatment (2 μM) for 24 and 48 h (Figure S1C). Results are shown as mean ± SEM (*$p \leq 0.05$, ** $p \leq 0.01$, *** $p \leq 0.001$).
